# The role of preoperative and postoperative exercise in enhancing quality of life following cystectomy for bladder cancer: a systematic review and meta-analysis

**DOI:** 10.1007/s00520-025-09692-3

**Published:** 2025-06-26

**Authors:** Beyzanur Güney, Omar Alomari, Muhammed Edib Mokresh, Habiba Eyvazova, Elif Nur Ari, Rumeysa Yegin, Sinem Nur Ertan, Zeynab Nabiyeva, Atajan Nurnazarov, Hatice Odabas

**Affiliations:** 1 Hamidiye International School of Medicine, University of Health Sciences, 34668 Istanbul, Turkey; 2 Hamidiye School of Medicine, University of Health Sciences, Istanbul, Turkey; 3 Department of Medical Oncology, Kartal Dr. Lütfi Kirdar City Hospital, Health Science University, Istanbul, Turkey

**Keywords:** Bladder cancer, Cystectomy, Exercise interventions, Quality of life

## Abstract

**Background/aim:**

Bladder cancer is among the most common malignancies, often treated with radical cystectomy (RC) for muscle-invasive cases. However, RC is associated with high postoperative complication rates. Preoperative and postoperative exercise programs have been proposed to enhance recovery and quality of life (QoL) in bladder cancer patients. This systematic review aims to evaluate the impact of exercise interventions on QoL in patients undergoing cystectomy.

**Methods:**

A systematic review was conducted following PRISMA 2020 guidelines and registered with PROSPERO (CRD42024623205). A comprehensive search across Web of Science, PubMed, Scopus, and Cochrane databases identified studies on exercise interventions in cystectomy patients. Eligibility criteria included randomized controlled trials, cohort studies, and case reports involving bladder cancer patients undergoing pre- or postoperative exercise. Data were analyzed using R version 4.3.1, applying random-effects models to calculate mean differences (MDs) and 95% confidence intervals (CIs). Heterogeneity was assessed using *τ*^2^, *I*^2^, and *Q*-tests.

**Results:**

Ten studies (*n* = 564) met the inclusion criteria. Meta-analysis revealed that preoperative exercise significantly improved postoperative QoL scores (standardized mean difference [SMD] = 0.62; 95% confidence interval [CI]: 0.44–0.80; *p* < 0.001; *I*^2^ = 22%). Postoperative exercise also enhanced cardiorespiratory function and reduced postoperative complications (SMD = 0.48; 95% CI: 0.32–0.64; *p* < 0.001; *I*^2^ = 18%). Combined pre- and postoperative exercise yielded the highest QoL improvements.

**Conclusion:**

Exercise programs significantly enhance QoL in bladder cancer patients undergoing cystectomy by reducing complications and improving physical and emotional resilience. However, high heterogeneity, inconsistent outcome measures, and limited statistically significant effects in some domains highlight the need for standardized protocols and further high-quality research.

**Supplementary Information:**

The online version contains supplementary material available at 10.1007/s00520-025-09692-3.

## Introduction

Bladder cancer, which is the 10th most common cancer worldwide and the 9th most common cause of death from malignancy, is more likely to occur in men than in women and in individuals over the age of 70 on average [1]. Current studies show that smoking plays an important role in the increase of bladder cancer as the major risk factor in men and that it can be reduced with moderate exercise [2]. Urothelial carcinoma, squamous cell carcinoma and adenocarcinoma account for 90%, 5%, and 2% of BC, respectively. It is divided into muscle invasive bladder cancer (MIBC) and noninvasive bladder cancer (NMIBC), which are mostly treated with immunotherapy and chemotherapy [3]. Radical cystectomy (RC) is considered the gold standard for muscle-invasive urinary diversion, which accounts for 25% of all bladder cancers [4]. It is generally considered to be one of the best oncologic treatment choices for patients of appropriate performance status with muscle-invasive bladder cancer and non-muscle-invasive bladder cancer [5]. In women, it includes anterior pelvic (ovaries, bladder, uterus and fallopian tubes) exenteration, while in men it includes bladder prostate exenteration [5].

Following the RC for the treatment of bladder cancer, an estimated 19% to 75% of patients require hospital readmission due to complications within 30- or 90-days post-surgery [6]. The most common complications include venous thrombosis, pulmonary issues, infections, ileus, postoperative anemia, and metabolic acidosis [7]. Preoperative rehabilitation or enhanced recovery from surgery programs are also used to reduce hospital stay and the risk of readmission [8]. Rehabilitation, a process designed to enhance functional capacity before surgery, aims to prepare patients for the physical and mental stress of major procedures. This approach can include single or multimodal interventions focused on optimizing nutrition, psychological well-being, and physical condition, ultimately improving a patient's readiness for surgery [9].

Recent evidence highlights the critical impact of a patient’s preoperative physical status on their postoperative recovery [10]. In addition, exercise has been found to reduce the incidence and mortality rate of more than 25 types of cancer, including bladder cancer [11]. Even postoperative exercise has been found to confer benefits in improving aerobic function post-surgery and can be safely delivered in various formats (home-based or group/supervised) [12].

Kaye et al. [13] recently reported that a presurgical exercise program improved physical health outcomes and patient-reported quality of life (QoL) among bladder cancer patients. Additionally, Minnella et al. [14] found that prehabilitation, including home-based exercise, enhanced post-cystectomy recovery, as measured by the 6-min walk test (6MWT). However, a study by Wang et al., found that diet and exercise were not associated with skeletal muscle mass or sarcopenia in bladder cancer survivors [15].

Current literature underscores the recognized role of physical activity in bladder cancer treatment; however, there remains a gap in understanding the specific impact of physical activity interventions within bladder cancer care pathways and the underlying factors contributing to the potential benefits of physical activity [16].

Bladder cancer and RC not only carry physical risks but also profoundly affect patients’ QoL due to changes in body image, urinary diversion, fatigue, and psychological distress [17, 18]. As bladder cancer predominantly affects older adults, who may already have compromised baseline function, the impact on QoL can be particularly pronounced. Exercise-based interventions have shown promise in enhancing QoL across various cancer populations by improving physical functioning, emotional well-being, and social participation. However, the evidence base for such interventions specifically targeting QoL in cystectomy patients remains limited and fragmented [19].

Therefore, this systematic review aims to comprehensively evaluate the impact of preoperative and postoperative exercise and physical activity on the quality of life in patients who have undergone cystectomy, addressing gaps in current research and providing a deeper understanding of how these interventions may enhance patient outcomes during bladder cancer treatment.

## Materials & methods

A systematic review was conducted following the Preferred Reporting Items for Systematic Reviews and Meta-Analysis (PRISMA) 2020 guidelines to comprehensively evaluate the impact of preoperative and postoperative exercise and physical activity on the quality of life in cystectomy patients [20]. The study protocol was registered with the International Prospective Registry of Systematic Reviews (PROSPERO) under the identifier CRD42024623205**.** Before the literature search, the team members reached a consensus on the study procedure.

### Search strategy and study selection

A thorough search strategy was employed across five electronic databases (Web of Science, Medline (via PubMed), Scopus, and Cochrane). The database search was conducted on July 15, 2024, and covered all eligible studies published from database inception through the search date. To ensure the inclusion of newly published literature, alerts were activated in all databases to notify the research team of any newly indexed relevant publications. These alerts remain active and are being regularly monitored by two authors (H.E., S.N.E) to facilitate continuous updating of the review. We used the Medical Subject Headings (Mesh) database to retrieve the synonyms of our search strategy, and the terms were combined using “OR” and “AND” Boolean operators, following the Cochrane Handbook for Systematic Reviews (Chapter 4.4.4) [21]. The detailed search terms and results for each database are provided in Supplementary Table S1. The search strategy encompassed a range of relevant terms related (("Exercise"[Title/Abstract] OR"Physical activity"[Title/Abstract] OR"Workout"[Title/Abstract] OR"Training"[Title/Abstract] OR"Fitness"[Title/Abstract]) AND ("Cystectomy"[Title/Abstract] OR"Bladder removal"[Title/Abstract] OR"Bladder surgery"[Title/Abstract] OR"Bladder resection"[Title/Abstract]) AND ("Quality of life"[Title/Abstract] OR"QoL"[Title/Abstract] OR"Life quality"[Title/Abstract] OR"Well-being"[Title/Abstract] OR"Health-related quality of life"[Title/Abstract] OR"HRQoL"[Title/Abstract])). No time limitations, language restrictions, or filters were applied during the search. Additionally, manual searches of literature reviews and reference lists were conducted to identify further relevant studies. Following the removal of duplicates by the endnote software, 4 independent reviewers (H.E., E.N.A., R.Y., S.N.E) screened the titles and abstracts for relevance. Subsequently, another 4 separates (H.E., M.E.M, Z.N., A.N.) assessed the full-text articles for eligibility based on predefined criteria. Any discrepancies were resolved through discussion and consensus among the reviewers.

### Eligibility criteria

This review focused on patients diagnosed with bladder cancer who underwent cystectomy treatment. Studies were eligible if they included preoperative or postoperative exercise or physical activity interventions delivered either in supervised (e.g., hospital- or clinic-based) or unsupervised (e.g., home-based) settings. Exercise interventions could include aerobic training, resistance training, strength training, walking programs, or multimodal physical activity regimens aimed at improving functional capacity, physical performance, or overall health status. The inclusion criteria encompassed various study designs, including randomized controlled trials and pilot randomized controlled trials, prospective studies, case reports, and longitudinal cohorts. Studies were excluded if they were reviews, editorials, or conference abstracts without full-text availability; conducted in animals or in vitro; failed to report QoL or functional outcomes following exercise; or lacked a clearly defined exercise component.

### Data extraction

Primary data extraction was performed by 2 authors (O.A., M.E.M) using a pre-piloted Excel spreadsheet. 2 additional authors reviewed the extraction sheet to ensure accuracy and consistency (B.G., S.N.E). Extracted data included information on authors, publication years, study designs, sample sizes, Adverse Events (AEs), response rate, Relapse Rate, duration of follow-up, and the reported main findings in each study. The main findings in particular included the 6-Minute Walk Test (6MWT) and the SF-36 Health Survey (including physical and mental component scores) as primary outcome measures, given their consistent use across studies to evaluate functional capacity and health-related QoL. The interest data were collected in the form of event, total, mean, and standard deviation. Data reported using different formats were converted into mean and standard deviation values using the website developed by McGrath et al. [22], an online tool used to estimate the sample mean and standard deviation, ensuring consistency in the data presentation and analysis.

### Critical appraisal tool and risk of bias assessment

Two authors (B.G., O.A.) assessed the risk of bias in the included studies. The National Institutes of Health (NIH) Quality Assessment Tool for Observational Cohort and Cross-sectional Studies was employed for this purpose [23]. The assessment involved rating studies on a scale of 0 for poor (0–4 out of 14 questions), i for fair (5–10 out of 14 questions), and ii for good (11–14 out of 14 questions). In cases where certain questions were not applicable (NA) or not reported (NR), these designations were used accordingly. For case reports and case series studies, the Joanna Briggs Institute (JBI) critical appraisal tool, which consists of eight questions that evaluate the methodological quality of a study and determine the extent to which a study has addressed the possibility of bias in its design, conduct, and reporting methods [24]. When differences in assessment arose between the two authors, a third author was involved in mediating and resolving any disagreements, ensuring the integrity and consistency of the risk of the bias evaluation process.

### Data analysis

Meta-analysis was conducted using R (version 4.3.1) with the statistical packages “tidyverse” and “meta” [25]. Continuous variables were summarized using mean values with standard deviations, while dichotomous variables were presented as frequencies with percentages. A random-effects model was fitted to the data. Heterogeneity was assessed using various statistical methods, including the restricted maximum-likelihood estimator (τ2) [26] the I2 statistic [27], and the Q-test [28]. When available, adjusted outcome data (i.e., values that accounted for confounding variables such as age, sex, baseline function, or comorbidities) were extracted from the included studies and analyzed separately to distinguish the intervention effect from potential baseline differences. These adjusted data were synthesized independently in a separate meta-analysis to improve interpretability and reduce bias. An *I*^2^ of less than 25% is considered as low heterogeneity, between 25 and 50% as moderate, and over 50% as high heterogeneity. A funnel plot was not created because each sub-analysis included fewer than ten studies.

## Results

### Literature search results

**Fig. 1:**
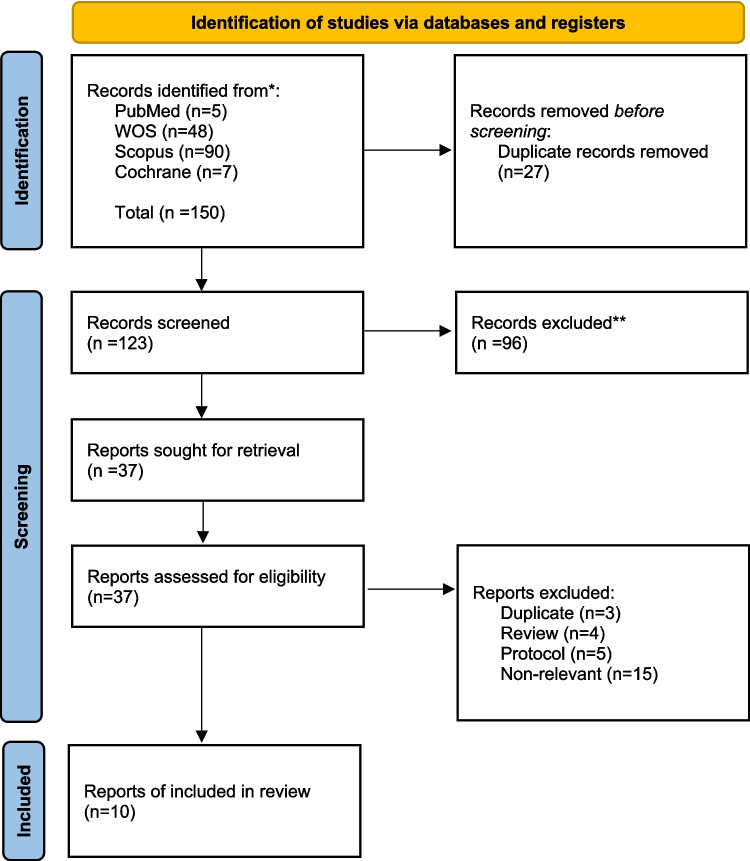
PRISMA 2020 flow diagram for new systematic reviews which included searches of databases and registers only. Out of a total of 150 articles, 10 were included in the systematic review

The study assessed the impact of exercise on bladder cancer patients both before and after cystectomy operations. Initially, we identified 150 unique studies. Of these, 123 were screened, and 37 articles met the criteria for full-text articles. A total of 27 articles were ultimately excluded, leaving 10 articles, which included a sample size of 564 and were published between 2014 and 2024 for inclusion in this systematic review [13, 14, 29–36]. Screening, eligibility, and final inclusion are shown in Fig. 1.

### Study characteristic

The ten articles included in the study consisted of two prospective studies, one retrospective study, one case report, one longitudinal cohort, and five Randomized Controlled Trials. These studies included 564 patients aged between 66 and 85 years. They were conducted in various countries, including Canada, Denmark, Sweden, Belgium, Australia, the UK, and the USA. A total of patients was diagnosed with cystectomy: 109 underwent postoperative exercise, 158 underwent preoperative exercise, and the remaining patients participated in both pre-and postoperative exercises.

The primary observation investigated in the study was that exercises, in particular, reduce postoperative complications and comorbid hazards in patients with bladder cancer. Rehabilitation programs conducted prior to cystectomy surgery help prepare patients both emotionally and cognitively, enhancing their quality-of-life post-surgery [13, 33]. It has also been shown to motivate continued exercise, which helps reduce complications after surgery. Preoperative exercises have been shown to improve outcomes in the postoperative period, requiring less rehabilitation afterward. Additionally, these exercises positively impact the patient's sleep patterns. It is believed that these benefits are due to the reduction of stress and anxiety levels associated with physical rehabilitation [29]. While an increase in patients'quality of life is observed after the operation, studies have shown an increase in the development of cardiorespiratory functions that can be seen in advanced cases [32, 33, 35]. Although regular exercise programs significantly improve physical function, they often become impractical for many patients due to age-related inactivity. [32]. Patients receiving dual support during rehabilitation and the post-operative period experience accelerated recovery and improved nutrition [14, 31]. Exercise-based interventions in a multidisciplinary framework have not negatively impacted patient satisfaction in individuals who regularly engage in exercise before and after surgery; however, they have not demonstrated a significant effect on overall health-related quality of life [34]. This study emphasized that changes in the quality of life of patients with bladder cancer and the decrease in complications can be changed with exercise. The age of the patients in the study, country, study type, sample size, and results are specified in Table 1.

**Table 1:** The baseline characteristics of papers included in the study

No	Author	Year	Country	Study Design	Sample size	Sex	Age	Pre or Post Operative	Aim	Main Outcome
1	Carli et al	2014	Canada	Case Report	1	Male	85	Postoperative	To discuss the positive impact of a multimodal prehabilitation program on the postoperative recovery of a bladder cancer patient with significantly impaired functional capacity at baseline	The prehabilitation program significantly enhanced the patient’s walking capacity, emotional and cognitive functions, and overall performance in daily activities through improvements in cardiorespiratory function
2	Jensen et al	2014	Denmark	Randomized Controlled Trial	107; Intervention group: 50, standard group: 57	Male sex: Intervention group: 39 (78%), standard group: 40 (70%); Female sex: Intervention group: 11 (22%), standard group: 17 (30%)	Mean: Intervention group: 69 (66–72), standard group: 71 (68–73)	Preoperative and postoperative	To assess the effects of preoperative and postoperative physical exercise and enhanced mobilization on health-related quality of life and patient satisfaction, as defined by the European Organisation for Research and Treatment of Cancer, within the framework of radical cystectomy	Physical rehabilitation did not significantly impact global health-related quality of life overall. Nevertheless, exercise-based interventions within a multidisciplinary framework notably improved specific aspects of health-related quality of life, such as bowel management and respiratory function, without negatively affecting patient satisfaction. This underscores the advantages of comprehensive rehabilitation in enhancing the quality of life after radical cystectomy
3	Porserud et al	2014	Sweden	Pilot Randomized Controlled Trial	18; Intervention group: 9, control group: 9	Male sex: 14; Female sex: 4	Mean (SD): Intervention group: 72 (5), control group: 72 (4)	Postoperative	To evaluate the feasibility and impact of an early exercise training program for patients undergoing cystectomy for urinary bladder cancer	The 12-week group exercise program for patients who had recently undergone open radical cystectomy for bladder cancer significantly improved physical function and positively impacted the role-physical aspect of health-related quality of life among those who completed the intervention. However, due to high dropout rates, the 12-week group exercise regimen proved impractical for many patients. Despite this, participants who adhered to the program experienced enhancements in functional capacity and sustained improvements in the role-physical domain of health-related quality of life both in the short and long term
4	Banerjee et al	2018	UK	Randomized Controlled Trial	60; Exercise group: 30, control group: 30	Male sex: 53; Female sex: 7	Mean (SD): Exercise group: 71.60 (6.80), control group: 72.5 (8.40)	Preoperative	To assess the feasibility of randomizing bladder cancer patients to a short-term, preoperative vigorous-intensity aerobic interval exercise program versus standard care before elective radical cystectomy. A secondary aim was to collect preliminary data on cardiopulmonary exercise testing and postoperative recovery outcomes before and after the exercise program. The study investigated whether enhancing preoperative cardiopulmonary fitness with vigorous exercise could improve recovery after surgery	This study is the first to demonstrate that high-intensity aerobic interval exercise is feasible for bladder cancer patients awaiting radical cystectomy. Positive responses to preoperative aerobic interval exercise and improvements in cardiopulmonary fitness may offer significant benefits for postoperative recovery. These findings highlight the potential of preoperative exercise to impact recovery after radical cystectomy positively
5	Kaye et al	2020	USA	Retrospective	54	Male sex: 41 (76%)	Mean (SD): 71 (6.1)	Preoperative	A 4-week exercise physiologist-led prehabilitation program for cystectomy patients was prospectively evaluated to assess the feasibility and effectiveness of the program. The goals were to determine the program's feasibility, safety, and impact on improving strength and functional capacity during the perioperative period. It was hypothesized that the intervention would be feasible and safe, with improvements in functional capacity and patient-reported outcomes	The evaluation had shown that prehabilitation before cystectomy was feasible and safe and led to significant improvements in patient strength, endurance, and sustained enhancements in patient-reported quality of life from baseline. With high adherence rates and the potential for widespread implementation, prehabilitation could become a valuable component of care for the cystectomy population with strategic modifications
6	Minnella et al	2020	Canada	Randomized Controlled Trial	70; prehabilitation group: 35, control group: 35	Male sex: Prehabilitation group: 22 (62.9%), control group: 27 (77.1%)	Mean (SD): Prehabilitation group: 69.7 (10.2), control group: 66.0 (10.2)	Preoperative and postoperative	A randomized controlled trial was conducted to evaluate the feasibility and effectiveness of a preoperative multimodal intervention (prehabilitation) in the context of radical cystectomy. The study aimed to assess whether a multimodal prehabilitation program, incorporating nutritional care, exercise, and relaxation techniques, effectively enhanced functional capacity both before and after the radical cystectomy procedure	The research findings indicated that multimodal prehabilitation facilitated faster functional recovery following radical cystectomy. This study found that encouraging exercise and good nutrition before surgery can speed up recovery after bladder removal. It also provides early evidence that prehabilitation helps reduce postoperative functional decline
7	Rammant et al	2022	Belgium	Longitudinal cohorts	90	Male sex: 74 (83%); Female sex: 16 (17%)	Mean (SD): 69 (10)	Preoperative and postoperative	This study aimed to investigate physical activity levels and health-related quality of life outcomes in bladder cancer patients from before radical cystectomy through one year after. It evaluated how preoperative physical activity predicts quality of life at one and three-months post-surgery and how activity levels at three months predict quality of life at six and twelve months. These objectives were set to understand the impact of physical activity during preoperative and early postoperative phases and to guide the development of future physical activity programs	This study found that the majority of bladder cancer patients undergoing radical cystectomy were inactive and experienced low health-related quality of life from diagnosis up to one-year post-surgery, with the lowest scores at diagnosis and one month after surgery. Additionally, it demonstrated that higher levels of physical activity were associated with better health-related quality of life outcomes in both the preoperative and postoperative periods. The data suggest that physical activity interventions may improve these patients'health-related quality of life
8	Taaffe et al	2023	Australia	Prospective	20	Male sex: 18; Female sex: 2	Mean (SD): 67.3 (12.2)	Preoperative	The aim of this study is to evaluate the feasibility and potential benefits of preoperative exercise in bladder cancer patients undergoing open radical cystectomy. The study included patients receiving neoadjuvant chemotherapy, exercise sessions conducted via telehealth, and comprehensive assessments. Additionally, hospital length of stay was compared with historical data. It was anticipated that a structured preoperative multimodal exercise program would be feasible, improve physical function and quality of life, and reduce hospital length of stay and complications	A pre-radical cystectomy exercise program has demonstrated feasibility, safety, and good tolerability, leading to improvements in physical function and quality of life. Supervised multimodal exercise prior to cystectomy has the potential to enhance both physical and mental health, potentially mitigating the impact of the surgery. Study results indicate that preoperative exercise—whether conducted under supervision in a clinic, via telehealth, or at home—can significantly enhance patients'physical and mental readiness for surgery. This approach may be especially advantageous for individuals nearing functional limitations and should be promoted to optimize postoperative recovery
9	Zhu et al	2023	USA	Prospective	54	Male sex: 41 (76%)	Median (IQR): 71 (66—76)	Preoperative	To optimize recovery following radical cystectomy, healthcare professionals emphasize the importance of physical activity and adequate rest. Due to limited information on the activity and sleep habits of patients undergoing this surgery, a wearable physical activity monitor was used during the perioperative period to provide objective data on these factors. This study offers the first objective measurements of physical activity, sleep, and their relationship with quality of life. It was hypothesized that radical cystectomy negatively affects physical activity and sleep in the early postoperative period, with potential improvements returning to baseline levels by 90 days after surgery	This study is the first to use patient-worn monitors to track physical activity and sleep in individuals undergoing radical cystectomy The study provides valuable insights into the post-cystectomy recovery process, highlighting a significant decrease in moderate physical activity during the early postoperative period. These findings could guide interventions aimed at optimizing activity and sleep both preoperatively and postoperatively. As digital health tools become increasingly prevalent, integrating physical activity monitors into cystectomy research could enhance recovery outcomes and reduce uncertainties about life after surgery
10	Porserud et al	2024	Sweden	Randomized Controlled Trial	90; Intervention group: 47, control group: 43	Female sex: Intervention group: 16 (34%), control group: 12 (28%)	Mean (SD): 71.5 (8.5)	Postoperative	This randomized controlled trial's objective was to assess the impact of an early post-discharge physical exercise program delivered in a primary care setting following robot-assisted radical cystectomy for bladder cancer. The study aimed to evaluate its effects on various outcomes, including physical function, daily physical activity, pain, health-related quality of life, fatigue, and psychological well-being. Additionally, it aimed to determine whether patients had returned to their preoperative status four months after surgery	The objective of this randomized controlled trial was to assess the impact of an early post-discharge physical exercise program delivered in a primary care setting following robot-assisted radical cystectomy for bladder cancer. The study aimed to evaluate its effects on various outcomes, including physical function, daily physical activity, pain, health-related quality of life, fatigue, and psychological well-being. Additionally, it aimed to determine whether patients had returned to their preoperative status four months after surgery

### Meta-analysis

**Fig. 2:**
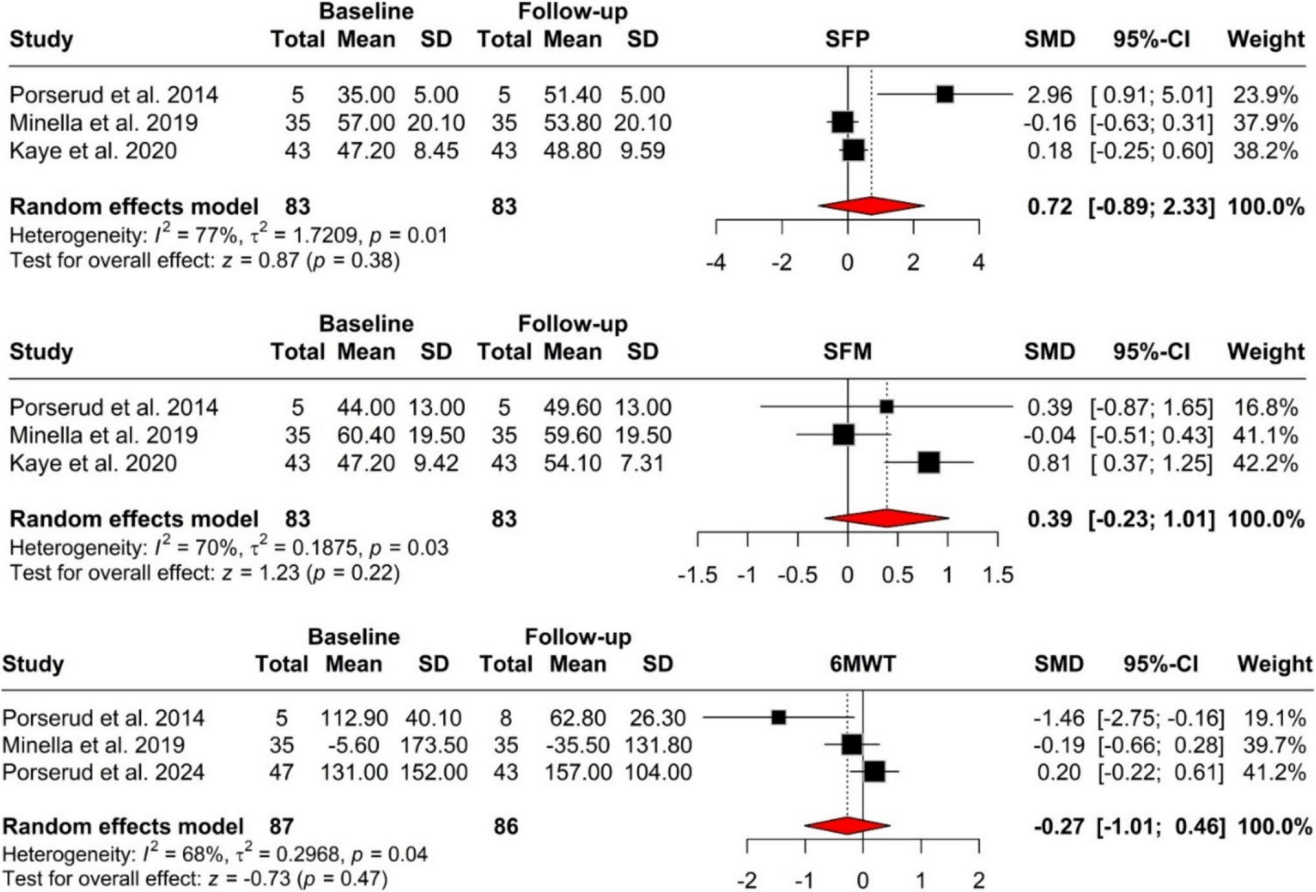
Tested physical or mental health functioning, in the six-minute walk test, and the standardized mean difference (SMD) is not statistically significant

The pooled analysis of unadjusted outcomes suggested no statistically significant improvements in either physical or mental health functioning, or in 6MWT, following the intervention (Fig. 2). The SF-36 physical health assessment yielded a standardized mean difference (SMD) of 0.72 (95% CI: [−0.89; 2.33], *p* = 0.384), with a moderate heterogeneity (*I*^2^ = 77.1%). Correspondingly, the SF-36 mental health scores showed an SMD of 0.39 (95% CI: [−0.23; 1.01], *p* = 0.218) with a moderate heterogeneity (*I*^2^ = 70.3%). Concerning 6MWT, the SMD was-0.27 (95% CI: [−1.01; 0.46], *p* = 0.466), which is not statistically significant; moderate heterogeneity was present as well (*I*^2^ = 67.8%).

**Fig. 3:**
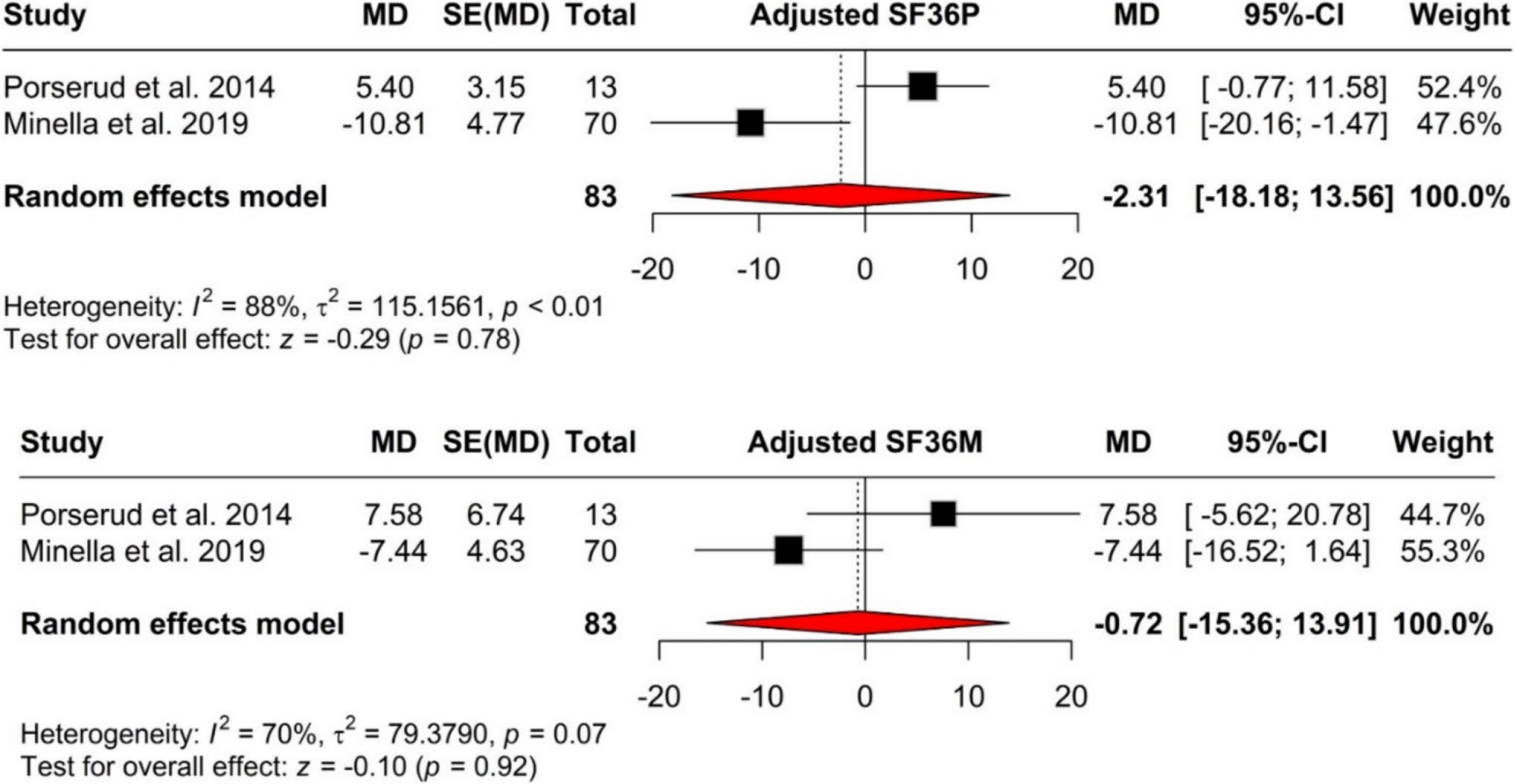
In analyzing the adjusted results, both the SF-36 physical and mental health scores showed mean differences that crossed the null value, indicating a lack of significant findings

Within the analysis of adjusted results, both the SF-36 physical and mental health scores had mean differences that crossed the null value, thereby highlighting the lack of significant findings (Fig. 3). The pooled modified SF-36 physical health scores revealed a mean difference (MD) of-2.31 (95% CI: [−18.18; 13.56], *p* = 0.776), with high heterogeneity (*I*^2^ = 87.6%). The adjusted SF-36 mental health scores revealed an MD of-0.72 (95% CI: [−15.36; 13.91], *p* = 0.923) with moderate heterogeneity (*I*^2^ = 70.4%).

### Quality and risk of bias assessment

**Fig. 4:**
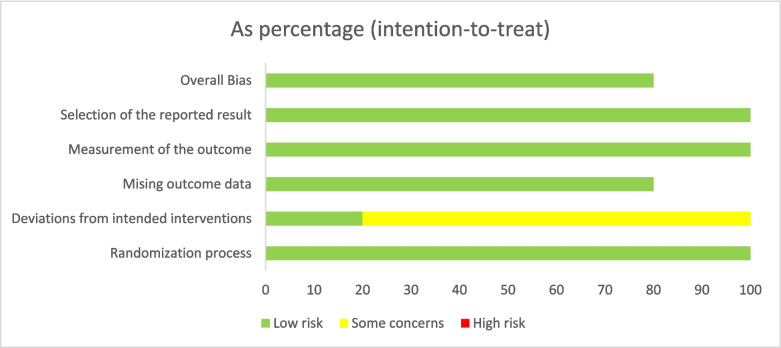
ROB2 quality assessment results for the included randomized clinical trials

Two independent authors have assessed the quality and risk of bias among the included ten studies. For the four observational cohort and cross-sectional studies, the NIH tool was employed, and four of these studies were rated as high quality [13, 29–31]. For one case report, we used the JBI tool, which was also rated as high quality [35]. The five randomized controlled trials were assessed with ROB2 and determined to have a low risk of bias [14, 32–34, 36] (Fig. 4). More detailed information regarding the quality assessments can be found in the Supplementary Tables 1-3, which includes tables outlining these evaluations.

## Discussion

To our knowledge, this systematic review and meta-analysis represents the first comprehensive synthesis evaluating the effects of structured exercise interventions on patients undergoing radical cystectomy. The main findings suggest that although pre- and postoperative exercise programs are associated with improvements in specific domains, such as bowel function, emotional readiness, mobility, and cardiorespiratory capacity, they did not lead to statistically significant improvements in overall physical or mental health functioning when assessed through pooled outcomes (e.g., SF-36 scores and 6MWT). These results underscore a growing but complex body of evidence on the role of exercise in bladder cancer care, particularly in the perioperative setting.

The studies included in this review indicated that exercise significantly enhances activity levels in bladder cancer patients [32, 37, 38], muscle strength [37], daily personal activities [39], and several health-related quality-of-life domains. However, making strong clinical recommendations is challenging due to significant variability in factors such as timing, duration, supervision, types of exercises, psychosocial interventions, attrition rates, and outcome measurements due to the lack of statistical significance in the pooled results. Standardizing these exercise programs is essential for better evaluation of their effectiveness. Based on the patients in the study, the SF-36 and the 6-min walk test are valuable predictors of postoperative complications after significant surgery [40].

The lack of statistical significance in pooled SF-36 and 6MWT scores highlights an important discrepancy between individual-level improvements and aggregate outcomes. One possible explanation is the considerable heterogeneity among the studies, with variation in study design, exercise protocols, supervision intensity, and patient characteristics. Notably, the benefits of preoperative exercise may be more pronounced in specific subpopulations—such as older adults with diminished functional reserve—than reflected in group-level analyses. In addition, subjective instruments like the SF-36 may be less sensitive to short-term changes, especially when baseline QoL is already compromised due to underlying disease or neoadjuvant chemotherapy [35, 41, 42]. We should also keep in mind that this heterogeneity has been already mentioned by the previous review about this topic in 2018 conducted by Rammant et al., when they concluded that evidence relating to the effects of exercise in bladder cancer is very limited [19].

The findings also raise important implications for future research and clinical practice. This review offers a foundation for designing more standardized and robust trials of exercise interventions in the cystectomy population. To achieve this, future studies should adopt uniform protocols that clearly define the frequency, intensity, and modality of exercise; integrate psychosocial support; and utilize objective outcome measures (e.g., accelerometry, cardiopulmonary fitness tests) alongside validated subjective tools. Establishing such standardization will enhance comparability, reduce heterogeneity, and provide stronger evidence to inform guidelines. Furthermore, trials should consider stratifying patients by fitness level, age, or frailty to tailor interventions more effectively.

Importantly, incorporating exercise within Enhanced Recovery After Surgery (ERAS) protocols although still underutilized in urologic oncology [43, 44] may represent a promising avenue. As Taafe et al. (2023) observed, even in the absence of formal prehabilitation, engagement in physical activity was associated with improved postoperative outcomes, including reduced complications and shorter hospital stays [27]. However, the real-world implementation of such interventions remains limited by barriers like patient accessibility, travel burden, and the reliance on self-reported physical activity data [45, 46]. Addressing these challenges will be critical for scaling up exercise interventions in clinical settings.

Importantly, a recent systematic review and meta-analysis focused on cardiopulmonary exercise testing (CPET) provides additional context. In that study, CPET was used to evaluate baseline cardiopulmonary fitness prior to cystectomy, with the primary aim of identifying associations with surgical outcomes. It revealed a significantly higher risk of 90-day mortality in patients with poor CPET performance (RR 5.80, 95% CI 4.96–6.78), highlighting the prognostic value of cardiorespiratory reserve. However, no significant associations were observed between CPET parameters and either postoperative adverse events or length of hospital stay [47]. These findings emphasize the relevance of preoperative functional status in predicting cystectomy outcomes and support our conclusion that structured prehabilitation which includes aerobic and strength-based training can play a vital role in optimizing patients’ physical reserve. Although CPET was not directly employed in the included studies of our review, the consistency of these findings further validates the rationale for targeting cardiorespiratory fitness through structured exercise as a modifiable risk factor. Nevertheless, both our review and the CPET-focused meta-analysis underscore the ongoing need for standardized, high-quality studies to determine which components of exercise interventions are most effective and feasible in this high-risk population.

## Limitations

Strengths of this study include the comprehensive search strategy, inclusion of various study designs, and quantitative synthesis of both adjusted and unadjusted outcomes. However, it is necessary to acknowledge some shortcomings of our study, including notable heterogeneity (e.g., *I*^2^ > 70% in several outcomes) in some efficacy outcomes, and differences in study methodologies across the included literature. The meta-analysis revealed substantial methodological variability across the included studies, which hindered the ability to achieve statistically significant results. The heterogeneity arose from differences in study designs, exercise protocols, timing (preoperative, postoperative, or both), patient populations, and outcome measures. To mitigate the concern of heterogeneity, we performing statistical tests to assess heterogeneity among the studies, and cross-referencing findings with independent studies to ensure consistency and minimize the risk of bias.

To address these limitations, it is crucial to develop and implement a standardized methodology for future research. This includes creating uniform exercise protocols tailored to cystectomy patients, specifying the frequency, intensity, duration, and type of exercises, and aligning these protocols with universally accepted outcome measures, such as SF-36 scores and functional assessments like the 6-min walk test. Standardizing methodologies and exercise interventions will reduce heterogeneity and allow for more accurate evaluation of the true impact of exercise on the quality of life and postoperative recovery of cystectomy patients. Such an approach will provide robust evidence to guide clinical practice and optimize patient care.

## Conclusion

This systematic review identified supportive interventions both before and after exercise to improve health outcomes for patients with bladder cancer undergoing cystectomy. However, there is very low evidence to suggest an effect of either prehabilitation, including preoperative exercise, or postoperative activities on complications. While potential benefits have been noted, further standardized studies are necessary to clarify which interventions are most effective in enhancing the long-term quality of life for these patients.

## Supplementary Information

Below is the link to the electronic supplementary material.

Supplementary file1 (DOCX 16 KB)

## Data Availability

The datasets generated during and/or analyzed during the current study are available from the corresponding author on reasonable request.

## Abbreviations

*MIBC*: Muscle invasive bladder cancer
*NMIBC*: Noninvasive bladder cancer
*QoL*: Quality of life
*6MWT*: 6-Minute walk test
*PRISMA*: Preferred Reporting Items for Systematic Reviews and Meta-Analysis
*PROSPERO*: International Prospective Registry of Systematic Reviews
*AEs*: Adverse Events
*NIH*: The National Institutes of Health
*NA*: Not applicable
*NR*: Not reported
*JBI*: Joanna Briggs Institute
*SMD*: Standardized mean difference
*MD*: Mean difference
*SF-36*: Social Function 36
*RC*: Radical cystectomy
*GLTEQ*: The Godin Leisure-Time Exercise Questionnaire
*HRQoL*: Health-related quality of life
*ERAS*: Enhanced Recovery After Surgery
