# Supplementary material for: The role of preoperative and postoperative exercise in enhancing quality of life following cystectomy for bladder cancer: a systematic review and meta-analysis
Source: Support Care Cancer. 2025 Jun 26;33(7):629. doi: 10.1007/s00520-025-09692-3 (PMC12198061; doi:10.1007/s00520-025-09692-3)
Supplement: Supplementary file 1 — Supplementary file1 (DOCX 16 KB) [file 520_2025_9692_MOESM1_ESM.docx]

**Table 1: Quality Assessment Tool for Observational Cohort and Cross-Sectional Studies (NIH)**

| ***Study*** | **Q1** | **Q2** | **Q3** | **Q4** | **Q5** | **Q6** | **Q7** | **Q8** | **Q9** | **Q10** | **Q11** | **Q12** | **Q13** | **Q14** | **Overall rating** |
| --- | --- | --- | --- | --- | --- | --- | --- | --- | --- | --- | --- | --- | --- | --- | --- |
| *Rammant et al.* | + | + | + | + | + | + | + | + | + | + | + | + | - | + | High Quality |
| *Kaye et al.* | + | + | + | + | + | + | + | + | + | + | + | NA | + | + | High Quality |
| *Taaffe et al.* | + | + | + | + | + | + | + | + | + | + | + | - | + | + | High Quality |
| *Montgomery et al.* | + | + | + | + | + | + | + | + | + | + | + | - | - | + | High Quality |

Quality Assessment Tool for Observational Cohort and Cross-Sectional Studies (NIH) (+): Yes/Low risk, (-): No/High risk, (*): Unclear, NA: Not applicable

**Table 2: JBI Critical Appraisal Checklist for Case Reports**

| **References** | **Q1** | **Q2** | **Q3** | **Q4** | **Q5** | **Q6** | **Q7** | **Q8** | **Overall rating** |
| --- | --- | --- | --- | --- | --- | --- | --- | --- | --- |
| *Carli et al.* | **+** | **+** | **+** | **+** | **+** | **+** | **+** | **+** | **High Quality** |

: Yes, (-): No, (*): Unclear, Not/Applicable (NA)

**Table 3: Cochrane Risk of Bias Tool for Randomized Controlled Trials (ROB2)**

| **References** | **Randomization process** | **Deviations from Intended Intervention** | **Missing outcome data** | **Measurement of the outcome** | **Selection of the reported result** | **Overall** |
| --- | --- | --- | --- | --- | --- | --- |
| *Jensen 2014* | + | + | + | + | + | + |
| *Porserud 2014* | + | * | + | + | + | + |
| *Banerjee 2017* | + | * | + | + | + | + |
| *Minnella 2019* | + | * | + | + | + | + |
| *Porserud 2024* | + | * | + | + | + | + |

Cochrane Risk of Bias Tool for Randomized Controlled Trials (ROB

(+): Yes/Low risk, (-): No/High risk, (*): Some concerns
